# Childhood Attachment to Pets: Associations between Pet Attachment, Attitudes to Animals, Compassion, and Humane Behaviour

**DOI:** 10.3390/ijerph14050490

**Published:** 2017-05-06

**Authors:** Roxanne D. Hawkins, Joanne M. Williams, Scottish Society for the Prevention of Cruelty to Animals (Scottish SPCA)

**Affiliations:** 1 Clinical and Health Psychology, School of Health in Social Science, University of Edinburgh, Medical School, Teviot Place, Edinburgh EH8 9AG, UK; jo.williams@ed.ac.uk; 2 Kingseat Road, Halbeath, Dunfermline KY11 8PQ, Fife, UK; gilly.ferreira@scottishspca.org

**Keywords:** attachment, attitudes, children, compassion, humane behaviour, pets

## Abstract

Attachment to pets has an important role in children’s social, emotional, and cognitive development, mental health, well-being, and quality of life. This study examined associations between childhood attachment to pets and caring and friendship behaviour, compassion, and attitudes towards animals. This study also examined socio-demographic differences, particularly pet ownership and pet type. A self-report survey of over one thousand 7 to 12 year-olds in Scotland, UK, revealed that the majority of children are strongly attached to their pets, but attachment scores differ depending on pet type and child gender. Analysis revealed that attachment to pets is facilitated by compassion and caring and pet-directed friendship behaviours and that attachment to pets significantly predicts positive attitudes towards animals. The findings have implications for the promotion of prosocial and humane behaviour. Encouraging children to participate in pet care behaviour may promote attachment between children and their pet, which in turn may have a range of positive outcomes for both children (such as reduced aggression, better well-being, and quality of life) and pets (such as humane treatment). This study enhances our understanding of childhood pet attachment and has implications for humane education and promoting secure emotional attachments in childhood.

## 1. Introduction

This study focuses on the under-researched topic of childhood attachment to pets. The aims are to examine socio-demographic differences in child-pet attachment such as age, gender, family affluence, and pet type, and to investigate associations between childhood attachment to pets and compassion, humane behaviour, and positive attitudes towards animals. This paper will begin with an overview of research on childhood attachment theory and examine why pets may create attachment opportunities for children.

### 1.1. Human Attachment and Attachment to Pets

Children are biologically pre-programmed to form emotional attachments with human caregivers to enable their survival [1,2,3,4]. Attachment refers to the innate ability to form bonds of affection and love toward others [4] and plays a significant role in infancy and later in life. Infants seek proximity, physical contact, and emotional connection with main caregivers for psychological, emotional, and physical support and protection [5,6,7,8]. Children’s early attachments allow them to treat their main caregiver as a safe base or secure base from which they can gain comfort and nutrition and also explore their environment [9]. These early experiences of human caring relationships create internal working models (IWMs) of emotional attachment that guide later human relationships. A secure attachment to human caregivers, typified by a synchronous relationship between child and caregiver, has a protective effect against psychopathology [10] and is related to empathy and prosocial behaviour development [11]. However, an insecure attachment to human caregivers can have long-term negative consequences for a child’s mental health, well-being, and behaviour and is a risk factor for anxiety disorders [12], delinquency [13], and animal cruelty [14]. Although human attachment research has been heavily influenced by ethological research and animal studies [1,15], the focus has been on a child’s emotional attachment to human caregivers and the subsequent impact this has on later human relationships and behaviour. Far less research has considered children’s attachments to pets and the importance of pet attachments for children’s development and their behaviour towards animals.

There is growing evidence that animals are capable of offering features of a secure attachment relationship for children and that children can form emotional attachment with pets that are consistent in some respects with human attachment theory [16,17]. Pets may offer children aspects of emotional attachment such as an affectional bond, special friendship, and may meet the prerequisites for an attachment relationship in terms of proximity seeking/maintenance, safe-haven, secure base and separation distress, which are observed in human-human attachments [16,17,18]. Pets may act as supplementary attachment figures satisfying many attachment functions, but are unlikely to fulfil all functions of secure human attachment relationships that develop between children and their caregivers. For example, the observed distress of children following separation from pets may not be due to children feeling less safe in a pets’ absence [17] but rather because of their concerns about their pets’ welfare [19].

Pets satisfy the need for comport and reassurance, assistance, and protection [20]. Attachments to a pet dog may function as a secure base by providing security and stability from which children can explore their environment. Furthermore, dogs may help children to regulate their emotions because they can trigger and respond to a child’s attachment related behaviour [17,18,19,20]. Insecure IWM of human attachment (due to difficult early experiences) may be a risk factor later in human relationships. However, pets may offer a pathway towards re-establishing attachment security with others, as found in children in foster care [17]. Pets can facilitate the development of human attachment relationships [21,22,23] and can act as another attachment figure in the absence or disruption of human attachment relationships, such as parental divorce [24,25].

Attachment theory can also be viewed from the perspective of the caregiver’s role. In their relationships with pet animals, children’s role of caregiving [26,27] might foster an attachment system with a pet animal. This may be activated by a bias towards infantile features (the ‘baby schema’) typical of many pet breeds [28]. This bias is often viewed as a reactive-phylogenetically ancient mechanism [29,30] that facilitates a nurturing reaction such as caregiving and protective behaviour. This process supports the development of the human-pet bond including anthropomorphism and motherese (infant directed speech to the pet) [31,32,33]. This bias towards infantile features is strongest amongst pet owners that display a strong attachment to their pets [34]. Furthermore, attachment difficulties between pets and their owners are similar to insecure attachments observed between children and caregivers, such as anxiety, avoidance, and negative expectations [35]. It should be noted that the research on pets’ attachments to human caregivers is based on work with adults and their pets, rather than pets’ attachment to children.

The evidence to date suggests that attachment to pets shares some features with human attachments [35,36] and children feel strong emotional connections to their pets [37]. There is also growing evidence that pet ownership and attachment to pets have a range of positive psychological, emotional, and physiological health outcomes for children and adults. These include: lower risk of depression [38], better quality of life [37,39], a greater sense of well-being [40], reduced psychological and physical distress, and reduced loneliness [24,41]. For example, attachment to dogs and cats is associated with higher quality of life among adolescents [39]. Other positive outcomes include extended social networks [42,43], and increased happiness, security, and self-worth [44]. These results may explain why animal assisted interventions are increasingly demonstrating potential success as alternative therapies [45,46]. The psychological benefits of attachment to pets have been found for a variety of pet animals, particularly dogs [47] and cats [48], possibly relating to the caring activities required by pet dogs and cats [39]. Psychological benefits have also been observed for smaller pets such as birds and rabbits [49], yet very little research has examined attachment to different pet types. Given the potential benefits of emotional attachments to pets, it is a priority for research to examine what factors promote attachment to pets and how attachment to pets might be fostered.

### 1.2. Direct Contact with Animals and Attachment to Pets

Direct contact with animals during childhood, such as caring for pets, may foster the development of attachment to pets. Caring for pet animals provides children with the experience of taking responsibility for another living being, may support the development of empathy, and has been shown to relate to more humane attitudes later in life [50]. Pets provide an opportunity for children to learn, practice, and become motivated to nurture another living being [51] and to practice perspective taking skills [52]. Pet care can potentially facilitate pet attachment development and foster a child’s moral development, as they begin to reason about issues of justice, kindness, fairness, and what is seen as morally ‘right’ with respect to animals and animal welfare [53,54]. Several studies have found a link between pet ownership (particularly dogs and cats), pet attachment, positive attitudes to animals, compassion, empathy, and prosocial behaviour [54,55,56,57,58,59]. Humane attitudes and concerns for animal welfare are related to a greater involvement in caring for pets during childhood [60] and similar results have even been found for virtual pets [61].

### 1.3. The Present Study

Recent research has highlighted the lack of studies into childhood attachment to pets. A question that remains unresolved is whether caring behaviour leads to attachment or whether children will care more for animals they are emotionally attached to. There is also a lack of research on the possible influence of socio-demographic factors on attachment to pets [62,63]. An expansion of research on the topic of childhood attachment to pets was therefore warranted. This study considers four research questions:

1. Are there socio-demographic differences in childhood attachment to pets?
2. Is pet ownership and pet type important in attachment between children and pets?
3. Does caring for a pet and compassion influence a child’s attachment to pets?
4. Are there associations between childhood attachment to pets and attitudes towards animals?

Based on extensive literature review, we hypothesised that there would be a range of socio-demographic influences that would influence the degree of attachment between children and their pets, including age, gender, family affluence, and pet type. We also hypothesised that strong attachment to pets would be associated with higher compassion to animals and pet care (caring and friendship) and positive attitudes towards animals.

## 2. Materials and Methods

### 2.1. Participants and Procedure

Participants included 1217 (51% boys, 49% girls) primary school children from 24 schools across Scotland, UK. Children were mostly aged between 7 and 12 years old (mean (M) = 9.7, standard deviation (SD) = 1, range 6.4–12.2 years) and came from two school year groups: Primary 4, 8–9 year-olds (52.8%, age M = 8.8, SD = 6, range 6.4–9.9 years); and Primary 6, 10–11 year-olds (47.5%, age M = 10.8, SD = 5, range 10–12.2 years). The majority of children had pets (67%) and had a pet of their own (54%). The types of pets recorded were: dogs (35%), cats (22%), small mammals (18%), fish/reptiles/amphibians (21%), birds (2%), and other (4%).

The ethical guidelines of the British Psychological Society, specifically relating to research with children, was adopted for this research and ethical consent was granted by the University of Edinburgh Clinical and Health Psychology Ethics Committee. Permission was sought from each local authority before schools were contacted. School participation was at the head teacher’s discretion and parental consent and child assent were obtained prior to data collection.

### 2.2. Questionnaire Measures

#### 2.2.1. Pet Ownership

These questions were adapted from the Childhood Pet Ownership Questionnaire [60] and relate to current and past ownership of pets, types of pets (none/dog/cat/small mammal/fish/reptiles/ amphibians/birds/other), the number of pets in the household, and whether there was a pet that the child considered to be their own [64].

#### 2.2.2. Family Affluence

The validated Family Affluence Scale (FAS) II [65] was included as a measure family wealth. This scale comprises of four questions: (1) Does your family own a car, van, or truck? (2) Do you have your own bedroom for yourself? (3) During the past 12 months, how many times did you travel away on holiday with your family? (4) How many computers does your family own? A composite FAS II score was calculated (range 4–13). For this study, we categorised children using a three-point ordinal scale using the following scoring; low family affluence (scores 4–7), middle affluence (scores 8–9), high family affluence (scores 10–13) (Cronbach’s alpha (α) = 0.333).

#### 2.2.3. Attachment to Pets

The Short Attachment to Pets Scale (SAPS) for Children and Young People, developed and validated by Marsa-Sambola et al. [41,45], was used to measure attachment to pets. One nine-item scale asked children to “please tell us how you feel about your favourite pet animal” with nine statements, e.g., “I love pets” and “I consider my pet to be a friend (or would if I had one)”. Each item was scored on a five-point Likert scale (“strongly agree” to “strongly disagree”). Total scores were calculated (minimum score 9, maximum score 45) (α = 0.85). This measure is based on extensive previous research and focuses on the strength of the emotional attachment of children to their pets. The measure includes items for ‘love and interaction’, ‘joy of pet ownership’, ‘affectionate companionship’, ‘equal family member status’, ‘mutual physical activity’, ‘pet problems’, and ‘general attachment’ (see [37,39]). It does not include some features of human attachment such as secure base behaviour.

#### 2.2.4. Attitudes towards Animals

The ‘attitudes towards animals’ measure [64] was adapted from the Pet Attitude Scale (PAS-M) [56,66]. This measure comprised three scales, each with various items scored on a five-point Likert scale (1—“strongly agree” to 5—“strongly disagree”). The first scale related to pet animals and comprised nine items (e.g., “all pet animals should be cared for by humans”). The second scale related to wild animals and comprised eight items (e.g., “wild animals should live free in the wild”). The third scale related to farm animals and comprised 12 items (e.g., “all farm animals should be able to go outdoors”). An overall total score for attitudes towards animals was calculated (minimum 28, maximum 140) (α = 0.72).

#### 2.2.5. Children’s Compassion towards Animals

The ‘compassion to animals for children’ (CCA) measure [64] comprised one five-item scale asking “what do you think about animals?” with five statements, e.g., “when I see an animal that is hurt or upset I feel upset” and “when I see an animal that is hurt or upset I want to help it”. The measure is scored on a five-point Likert scale (“strongly agree” to “strongly disagree”). Total scores were calculated (minimum score 5, maximum score 25) (α = 0.58).

#### 2.2.6. Children’s Reported Humane Behaviour towards Animals (CRHBA)

The ‘humane behaviour’ measure [64] was adapted from a combination of the Children’s Treatment of Animals Questionnaire [11] and the Lexington Attachment to Pets Scale [67]. One 12-item scale asked children “how often do you do the following things with or for your pet animal(s) (or would if you had one)?” for each of 12 statements, e.g., “play with”, “cuddle”, and “talk to”. Each statement is scored on a scale of 1 to 4 (“often”, “sometimes”, “never”, and “I cannot do this with my animal”). Total scores were calculated (minimum score 12, maximum score 48) (α = 0.84).

Principal components analysis (PCA) extracted three components from the humane behaviour variables explaining 58.7% of the overall variance (Table 1). Component one, explaining 31.11% of the variance, was labelled “caring behaviour towards animals”. Component two, explaining 18.35% of the variance, was labelled “friendship behaviours to animals”. Component three, explaining 9.23% of the variance, was labelled “aggression towards pets”. As component three only had one item (“shout at”), it was excluded from further analyses. The two reliable subscales are used in subsequent statistical analysis presented below.

### 2.3. Procedure

All children completed the questionnaire within their school classroom (approximately 15 min to complete). Questionnaires were administered to the children during class time by school teachers (following standardised instructions). Each child completed the questionnaire individually at their classroom desk and could ask for help from a teacher if they had difficulty reading or understanding any of the questions. Teachers were instructed that they could help children read questions and answer on procedural queries, but they could not interpret questions or advise children on how to answer. The questionnaire used appropriate terminology for the age range and a pilot study with three schools (*N* = 128) confirmed its suitability. The questionnaires were either mailed or hand delivered to schools, following completion the questionnaires were sealed in an envelope and either collected in person or sent by mail and then stored securely within the University of Edinburgh. All information is treated confidentially and kept secure at all times; child and school data have been anonymised during data preparation by adopting identity numbers.

## 3. Results

Total scores were calculated for each key variable for each individual and data was analysed using SPSS Statistics 22 (SPSS Inc., Chicago, IL, USA), with a two-tailed significance of *p* < 0.05.

### 3.1. Descriptive Statistics

The majority of children displayed high attachment to their pets, or would if they had pets, with 69.2% scoring over the mean for total attachment. Children with pets scored higher on attachment for all of the items (Table 2).

### 3.2. Are There Socio-Demographic Differences in Childhood Attachment to Pets?

#### 3.2.1. Age and Gender

The results of *t*-tests found that girls scored significantly higher on attachment to pets than boys across all ages (t(1159) = 5.06, *p* = 0.000, *d* = 0.3). Girls also scored higher on compassion (t(1109) = 4.04, *p* = 0.000, *d* = 0.24), friendship behaviour (t(1135) = 7.16, *p* = 0.000, *d* = 0.43), caring behaviour (t(1139) = 2.53, *p* = 0.012, *d* = 0.15), and attitudes to animals (t(1032) = 2.88, *p* = 0.004, *d* = 0.18) than boys (Table 3).

There was no significant difference in attachment to pets between younger (6–9 years) and older children (10–13 years) (t(1112) = 0.17, *p* = 0.87, *d* = 0.02). Older children did, however, score higher on caring behaviour (t(1095) = 3.37, *p* = 0.001, *d* = 0.2) but not compassion (t(1077) = 1.5, *p* = 0.13, *d* = 0.09), friendship behaviour (t(1093) = 1.46, *p* = 0.145, *d* = 0.09), or attitudes (t(971) = 0.46, *p* = 0.64, *d* = 0.03) (Table 3).

#### 3.2.2. Family Affluence

One-way ANOVA found no significant difference between categories of family affluence for attachment scores (F(2,1142) = 1.73, *p* = 0.18, *n*^2^ = 0.003). An initial significant difference in attitudes to animals scores was found (F(2,1014) = 3.8, *p* = 0.04, *n*^2^ = 0.01), but not following post-hoc analysis. No significant difference was found between categories of family affluence for compassion (F(2,1102) = 0.66, *p* = 0.52, *n*^2^ = 0.001), friendship behaviour (F(2,1127) = 1.73, *p* = 0.18, *n*^2^ = 0.003), or caring behaviour (F(2,1127) = 1.04, *p* = 0.35, *n*^2^ = 0.002).

### 3.3. Is Pet Ownership and Pet Type Important in Attachment between Children and Pets

#### 3.3.1. Pet Ownership and Attachment to Pet Scores

Following one-way ANOVA analysis a significant difference in attachment scores was found between groups of children based on the number of pets they owned (F(3,1153) = 15.9, *p* = 0.000, *n*^2^ = 0.04). Children without pets scored significantly lower on attachment than children with one (*p* = 0.000), two (*p* = 0.000), and more than two pets (*p* = 0.000). The results of *t*-test analyses found that children who had a pet in their past or currently had a pet of their own scored significantly higher on attachment (Table 4).

#### 3.3.2. Pet Type and Attachment to Pet Scores

Descriptive statistics (Table 4) shows that children with dogs scored the highest on attachment, followed by cats, small mammals, other (e.g., horse), fish/reptiles, or amphibians, and that children with pet birds scored the lowest on attachment. The results of *t*-test analyses showed that children who had a pet dog or cat scored significantly higher on attachment than children who did not (Table 4). There was no significant difference in attachment scores between children who had or did not have small mammals, fish/reptiles/amphibians, birds, or other pet animals.

Multiple regressions using the step-wise method were run to predict attachment to pets from ownership of pet types. Dogs (*p* = 0.000) and cats (*p* = 0.000) statistically predicted attachment scores (F(2,1158) = 66, *p* = 0.000, *f*^2^ = 0.11) together explaining 10.2% of the variance in attachment scores. All other pet types were insignificant (*p* > 0.05) and so were removed from the model.

#### 3.3.3. Relations between Pet Care, Pet Attachment, Compassion, and Attitudes to Pets

Regression analyses were conducted to establish whether there were associations between scores for attachment to pets and caring behaviour, friendship behaviour, compassion, and attitudes. We examined attachment scores as both an independent variable (IV) and dependant variable (DV) in separate analyses to give us an indication of the direction of these associations. By examining the beta (B) regression coefficients, we assessed the strength of the relationship between each predictor variable (IV) to the criterion (DV), with the higher the beta value (displayed in bold in Table 5), the stronger the relationship.

### 3.4. Does Caring for a Pet and Compassion Influence a Child’s Attachment to Pets?

**Figure 1:**
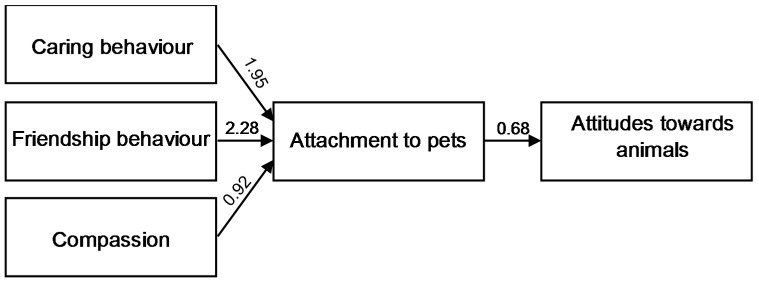
The relationship between attachment to pets and caring behaviour, friendship behaviour, compassion, and attitudes. Unstandardized regression coefficients (ß) are included as demonstrators of strength of the direction. All *p* < 0.005.

Results from the regression analyses (Table 5) show that caring behaviour and friendship behaviour significantly predicted attachment to pet scores. Attachment to pet scores increased for every 1.95 point increase in caring behaviour, and increased for every 2.28 point increase in friendship behaviour. Caring for pets and friendship behaviour towards pets are therefore important facilitators of attachment to pets. Compassion towards animals also significantly predicted attachment scores. Attachment scores increased for every 0.92 point increase in compassion. Compassion towards animals is therefore important for facilitating attachment to pets. A simple diagram demonstrating the strength of these associations is shown in Figure 1.

### 3.5. Are There Associations between Childhood Attachment to Pets and Attitudes towards Animals?

Results from the regression analyses (Table 5) show that attachment to pets significantly predicted positive attitudes towards animals. Attitude scores increased for every 0.68 point increase in attachment scores. Attachment to pets is therefore important for facilitating positive attitudes towards animals (see Figure 1).

## 4. Discussion

The aim of this study was to investigate the relationships between childhood attachment to pets, pet care, compassion to animals, and attitudes towards animals. We first examined socio-demographic factors and focused particularly on pet ownership and types of pets owned. We found that the majority of children scored high on attachment to pets, but these attachment scores differed depending on pet ownership, pet type, and gender of the child. We found associations between attachment to pets and caring behaviour, friendship behaviour, compassion, and attitudes, and examined the direction of these relationships. We will begin by discussing the findings in detail before considering the implications and limitations of the study.

Our results demonstrate that children (at least in this Scottish sample), are highly attached to their pets. The mean score for attachment was 15 (score of 9 was highest and 44 was lowest attachment score possible) and 69.2% of the children scored below 15. Pets are important in children’s lives, with 80% of our sample reporting that they loved pets, 83% of those with pets reported that their pet made them happy, 76% reporting that their pet was their best friend, 62% reporting that they would be lonely without their pet, and 52% reporting that they felt that their pet knew when they were upset and it tried to comfort them. These findings support previous research demonstrating the significance of pets in the lives and social networks of children, that they form close emotional connections to their pets, and that pets may provide a source of affection and comfort [39,68,69]. Children are emotionally expressive towards their pets and are strongly connected to them, often reporting them as one of the most important figures in their lives [70]. Both the current study and previous work therefore demonstrate that pets are important source of emotional attachment in the lives of children and support the notion that pets should not be overlooked in attachment research.

When we examined strengths of the associations between attachment to pets and caring and friendship behaviour, compassion, and attitudes towards animals, we found that caring behaviour, friendship behaviour, and compassion were significantly associated with attachment scores. Attachment to pets was significantly associated with attitudes towards animals. The finding that caring behaviours (such as spending time with pets, cuddling, stroking, and playing with pets) and friendship behaviours (such as telling secrets to, crying with when sad, and talking to pets) were significantly associated with attachment to pets is consistent with previous findings [71]. Although we cannot ascertain causation, these findings suggest that children’s participation in pet caring roles at home may possibly foster attachment to their pets, which may have positive outcomes for the child (e.g., improved well-being and quality of life [37,38,39]) as well as the animal (e.g., better care and welfare [27,37,72]). From direct experience, children learn to be nurturing, and develop the ability to recognise, understand, and share the feelings of others [73,74]. This may also explain why attachment to pets is related to children’s beliefs about animal minds [64], the attribution of emotions to pets [75], and predicts a more reliable and consistent ability to interpret animal behaviour and facial expressions [76,77]. The finding that compassion towards animals (such as feeling upset and wanting to help when an animal is hurt or upset) is associated with attachment scores, suggests that children’s attachment to their pets is connected with empathetic and compassionate orientations towards pets. Again, although we have not directly tested causation here, this makes sense, as those who are securely attached to others are more likely to develop compassion in their relationships [78]. Humane education or other activities that facilitate empathy and compassion could therefore potentially promote positive attachment to pets. Promoting compassion and empathy towards animals has important implications for prosocial behaviour towards other children, as animal-directed empathy can generalise to human-directed empathy [79,80].

Furthermore, we found that attachment to pets significantly predicted positive attitudes to animals, and from previous research we know that attitudes are associated with empathy, pro-social, and humane treatment of animals, greater concern for animal welfare, and less cruelty [11,59,81]. Our findings support previously identified links between pet attachment, empathy, positive attitudes to animals, and a prosocial orientation and behaviour [58,59,82,83]. Attachment to pets is a relational concept rather than an attitude or ideology [84]. High attachment to pets can promote positive child-pet relationships, whilst low attachment to pets may have a negative impact on child-pet relationships [35]. High attachment facilitates nurturance and humane behaviour, whereas low attachment has been related to higher acceptance of animal cruelty [14]. Animal cruelty and neglect may be associated with a lack of emotional attachment between child and their pet.

Considering socio-demographic factors in attachment to pets, we found that girls were significantly more attached to their pets than boys. Girls also scored significantly higher on caring behaviour, friendship behaviour, and compassion, which supports previous findings in other studies of children [37,56,83] and adults [85]. However, other studies have found no gender difference in pet attachment [63] or in care related behaviour towards pets [86]. These mixed findings may be explained by different populations and measurement tools. We found no significant difference in attachment scores between low, medium, or high family affluence, which is consistent with previous findings that also found no difference [37], although Westgarth et al. [63] found that deprivation increases with the number of dogs owned. However, the internal reliability of the Family Affluence Scale used in this study was low and results should be interpreted with caution. This measure is widely used in child health research and has been developed specifically as a child and adolescent self-report research tool. We also found no significant difference in attachment to pets between younger and older children, however, research with adolescents shows that attachment to pets decreases between 11 and 15 years of age [37]. It may therefore be worth investigating attachment to pets in a wider age range in the future. We did find, however, that older children scored higher on caring behaviour, possibly due to increased responsibility for pet care within the home [27].

Our findings show that children with pets (or who had pets in their past) scored higher on all attachment items than children without pets or those who had never had a pet. These findings suggest that children who grow up with pets have an early opportunity to form pet attachments, become emotionally connected to their pets through direct experience, spend quality time with their pets, and experience a ‘sharing of significant moments’, which is consistent with previous research [37,39,57,87]. For example, children view pets as confidantes for secrets, demonstrating this emotional connection [69]. Although pet ownership alone is important, it seems to be that having a pet that a child feels is their own has the most influence, as we found that children with their own pet scored higher on pet attachment. This finding supports the argument that a close relationship and emotional bond is more important than merely the presence of a pet within a home [82], possibly due to increased direct involvement and responsibility for the care of their pet, thus facilitating attachment.

In relation to pet type, we found that the type of pet that a child has influenced the degree of child-pet attachment. Children with pet dogs scored the highest on attachment, which makes sense given children demonstrate high attachment to dogs, view dogs as attachment figures, and have more direct contact with dogs inside and outside of the home [63,71]. Dogs are also more likely to read and adapt their behaviour in response to human emotional signals [88]. Children also demonstrated high attachment to cats, followed by small mammals, fish/reptiles/amphibians, and other (e.g., horse), with the lowest scores being shown for birds. Pet dogs and cats were the only significant predictors of child-pet attachment. The finding that higher attachment is shown for dogs and cats is consistent with previous research in adults [89] and children [83] and may be explained by the wider range of behaviours that can be displayed between children and their pet dog or cat compared to other animals. Dogs and cats may also be more receptive to our emotions and express their emotions and behaviour more clearly than other pet animals, facilitating a closer connection or bond. However, most attachment research uses measures that are based on direct interactions with pet dogs (e.g., groom and walk) rather than emotional aspects (e.g., joy and love) that can be expressed for all pets [89], which may explain these findings.

### Limitations and Future Directions

This was a large-scale questionnaire study using self-report data and thus might be subject to response biases such as social desirability, potential peer influence, and demand characteristics. Although we did not measure behaviour directly there is a strong evidence base for the link between attitudes and behaviour [90]. Self-report questionnaire methods are a tried and tested approach for children of this age range, but it is possible that a minority of the younger children included in the sample may have needed some teacher support in completing the questionnaire (provided as part of the data collection procedure). However, only two children were under the age of 7 years and teachers were instructed to help children only with reading items and were discouraged from interpreting items or suggesting answers to minimise teacher effects. Future research would benefit from using a combination of self-reports, parent reports, observational, and behavioural methods to allow data triangulation and ensure accuracy of findings.

There are also some limitations with the measures used in this research. In the humane behaviour measure, the sub-scale “aggression towards pets” consisted of only one item and was excluded from analysis, however, we know from previous studies that low attachment to pets is related to cruelty to animals [14]. The SAPS, used to measure pet attachment, was highly reliable within our sample and in those which validated its use [37,39,62] and is a short measure of attachment developed for use in large survey research. It should be noted that it does not cover all aspects of attachment relationships, focusing more on the emotional aspects of pet attachment such as ‘crying with’ (comfort) and feeling lonely without them (separation anxiety) and the friendship aspects of pet attachments, such as playing with and spending time with their pet every day. Other aspects of attachment relationships (e.g., secure base behaviour) are not included, which is a potential limitation. To enable all children to answer the SAPS they could answer about a pet they had or a hypothetical pet (this could be a pet they used to have or one owned by a relative). Our research indicates differences in attachment between children who currently have pets and those who do not. Further analysis could explore in more depth differences in pet attachment between current pet owners and those who currently do not have a pet, but perhaps have had a pet previously, wish to own a pet, or have a strong connection to a pet owned by someone else (relative, friend, or neighbour). It would also be interesting to look at differences between how children respond to the items within SAPS in relation to different pets (e.g., dog owners and cat owners [91]). In terms of the analysis exploring associations between pet care and attachment, it should be noted that two items in SAPS (“I spend time every day playing with my pet” and “I talk to my pet quite a lot”) are similar to those on the pet care measure (such as “play with” and “talk to”) and this may have influenced the findings. Despite these limitations, SAPS provides interesting insights into children’s attachment to pets, and is linked to positive outcomes such as quality of life [37,39] and humane behaviour (as seen in the current study). It would be interesting to use this measure in samples outside the UK to examine its cross-cultural reliability and to complement SAPS with other measures of pet attachment using mixed methods approaches. Although research on attachment to pets is expanding, there are still inconsistent results [92] which may be due to the use of different measures of attachment. Developing and refining age-appropriate measures for child-animal interaction research remains a priority for research.

In a short survey it is not possible to capture data on all variables of potential interest. In this study we did not consider family dynamics such as dual or single-parent families and sibling status, which could have influenced attachment scores (children in single parent families and youngest children show greater attachment to their pets [63,93]). Although we used a diverse sample from across Scotland that included a variety of ethnicities and religions we did not include measures of ethnicity, religion, or cultural background, which have been shown to influence human-animal interactions [85]. Future research might also consider the impact of pet loss and grief as an indicator of pet attachment and the impact it may have on children’s development and mental health, including anxiety and depression [94,95].

## 5. Conclusions

This study examined the emotional and friendship aspects of children’s attachments to pets and found that attachment to pets was associated with children exhibiting caring and friendship behaviours to pets, and compassionate views towards animals. The findings have implications for the promotion of prosocial and humane behaviour. Although causation cannot be inferred from cross-sectional data, the strong statistical associations between pet attachment and caring behaviour found here suggest that encouraging children to participate in pet care behaviour may have a range of positive outcomes for both children (such as better well-being, quality of life) and pets (such as better welfare and humane treatment). Overall, this study enhances our understanding of childhood attachment to pets, but further research is required to examine how and when children form attachments to animals and how we might promote positive attachment to pets. We also need to consider the long-term mental health outcomes for different child-pet attachment types and whether attachment to pets might facilitate secure attachment in children’s human relationships.

## Figures and Tables

**Table 1:** Principal component analysis with varimax rotation showing variable loadings on each component extracted from analyses (*N* = 1217). High loadings are in bold.

Sub-Scale	Item from the Children’s Reported Humane Behaviour toward Animals (CRHBA) Measure	Component		
		1	2	3
Caring Behaviour	Pat/Stroke	**0.835**	0.154	0.179
	Cuddle	**0.821**	0.245	0.064
	Play	**0.777**	0.240	0.084
	Take for a Walk	**0.645**	0.074	–0.371
	Groom (Comb Hair)	**0.642**	0.336	–0.142
	Spend Time with	**0.613**	0.391	0.229
	Allow to Stay in Room	**0.509**	0.192	0.032
Friendship Behaviours	Tell Secrets to	0.189	**0.779**	–0.111
	Give Food or Water to	0.093	**0.645**	0.040
	Cry with When Sad	0.366	**0.609**	–0.025
	Talk to	0.341	**0.599**	0.236
Aggression	Shout at	0.075	0.027	**0.886**

Note: the sub-scale ‘aggression’ was removed in future analyses. Bold numbers highlight each items’ best fit within the components.

**Table 2:** Descriptive statistics for children’s attachment to pets for all children and pet owners.

		Strongly Agree (%)	Agree (%)	Not Sure (%)	Disagree (%)	Strongly Disagree (%)
I don’t really like animals (reverse coded)	All	3.6	2.2	4.8	14.2	75.2
	Pets	2.9	1.2	2.4	10.5	83
I spend time every day playing with my pet (or would if I had one)	All	50.2	31.8	11.2	4	2.8
	Pets	50.8	32.3	10.5	4.1	2.4
I have sometimes talked to my pet and understood what it was trying to tell me (or would if I had one)	All	36.4	31.7	19.4	6.3	6.2
	Pets	40.2	33.7	14.9	6.2	4.9
I love pets	All	79.5	12.9	4.1	1.3	2.2
	Pets	86.5	8.8	2.5	0.2	2
I talk to my pet quite a lot (or would if I had one)	All	44.4	30.1	13.7	7	4.7
	Pets	48.4	29.8	10.7	6.9	4.1
My pet makes me feel happy (or would if I had one)	All	77.9	15.2	3.8	1.2	2
	Pets	83.2	12.6	1.9	0.7	1.6
I consider my pet to be a friend (or would if I had one)	All	73.4	18.3	4.4	1.8	2
	Pets	76.1	16.1	4	1.9	2
My pet knows when I am upset and tries to comfort me (or would if I had one)	All	46.8	21.1	22.9	4.9	4.3
	Pets	51.9	20.6	18.4	5.1	4
There are times I’d be lonely without my pet (or would if I had one)	All	58.2	22.9	11	3.6	4.4
	Pets	62	23.5	7.9	3	3.6

**Table 3:** Descriptive statistics for socio-demographic variables.

	Attachment (Strong 9 → 44 Weak)		Compassion (High 5 → 25 Low)		Friendship Behaviour (High −3 → 4 Low)		Caring Behaviour (High −2 → 4 Low)		Attitudes (High 31 → 94 Low)	
	M	SD	M	SD	M	SD	M	SD	M	SD
Girls	14.17	6.4	7.83	2.8	−0.21	0.92	−0.08	0.95	49.4	9
Boys	15.91	6.4	8.56	3.3	0.20	1	0.07	1	51	10
Primary 4	14.93	6	8.04	3	−0.05	1	0.09	1	50.28	10
Primary 6	14.99	5.7	8.31	3	0.04	1	−0.11	1	50.01	9
Low FA	16.02	7	8	3	0.09	1	0.09	1	51.79	11
Medium FA	15.24	6	8.05	3	−0.09	1	0.05	1	51.11	9
High FA	14.85	6	8.27	3	0.02	1	−0.03	1	49.7	9.3

Note: FA: family affluence. M: mean; SD: standard deviation.

**Table 4:** Results from *t*-tests examining differences in attachment scores for pet ownership.

	Yes		No		Result			
	M	SD	M	SD	df	t	*p*	*d*
Own Pet	14	5	16.3	6.5	993	−6.9	**0.000**	0.4
Pet in the Past	14.4	5.5	17.4	7	1147	−7.15	**0.000**	0.5
Dog/s	12.9	4.4	16.3	6.3	1109	10.9	**0.000**	0.63
Cat/s	13.8	5.1	15.4	6.1	485	4.3	**0.000**	0.19
Small Mammals	14.55	5.3	15.2	6.1	341	1.5	0.14	0.11
Fish/Reptiles/Amphibians	15.2	6.4	15	5.8	1158	−0.26	0.798	0.02
Birds	16.4	8.3	15	5.9	1158	−1.1	0.27	0.2
Other	15.1	5.9	14.7	7.2	1158	0.44	0.66	0.06

Note: Low score = high attachment.

**Table 5:** Results from regression analysis for attachment to pets.

	Result			Attachment as IV			Attachment as DV		
	df	F	*p*	R	VE %	B	R	VE %	B
Attitudes	1000	237	0.000	0.44	19.2	**0.68**	0.44	19.2	0.28
Compassion	1080	304	0.000	0.47	22	0.24	0.47	22	**0.92**
Friendship behaviour	1107	191	0.000	0.38	14.7	0.06	0.38	14.7	**2.28**
Caring behaviour	1107	131	0.000	0.33	10.6	0.06	0.33	10.6	**1.95**

Note: IV: independent variable; DV: dependent variable; VE: variance explained; B: Unstandardized regression coefficients; bold: indicates strength of the direction.
